# Ferroptosis-Related Prognostic Gene LAMP2 Is a Potential Biomarker Differential Expressed in Castration Resistant Prostate Cancer

**DOI:** 10.1155/2023/8295113

**Published:** 2023-01-20

**Authors:** Chuanyu Sun, Ningning Zhang, Qingfeng Hu, Guowei Xia

**Affiliations:** ^1^Department of Urology, Huashan Hospital, Fudan University, Shanghai, China; ^2^Department of Anesthesiology, Huashan Hospital, Fudan University, Shanghai, China

## Abstract

**Background:**

It remains unclear about the mechanisms of prostate cancer progressing to castration resistant prostate cancer (CRPC) and the correlation with ferroptosis.

**Methods:**

We compared the gene profiles between localized prostate cancer and metastatic CRPC using the GEO dataset and intersected with a cluster of known ferroptosis-related genes. We received differentially expressed genes (DEGs) in CRPC related to ferroptosis and performed survival analysis to analyze the prognostic values. Furthermore, we conducted single sample gene set enrichment analysis (ssGSEA) to analyze immune infiltration and investigate microRNA crosstalk and methylation for prognostic genes using online databases.

**Results:**

We identified 84 DEGs in CRPC related to ferroptosis and 19 hub genes densely connected into networks by enrichment analysis. We performed survival analysis and Cox regression for these genes and identified LAMP2 with significantly prognostic values in overall survival (OS) and disease-specific survival (DSS) of prostate cancer. Furthermore, we found immune infiltration of various immune cells significantly correlated with LAMP2 expression in prostate cancer and identified multiple microRNAs associated with LAMP2 expression in prostate cancer. In addition, we found that the methylation level of LAMP2 in prostate cancer was significantly associated with cancer and identified 8 methylation sites for LAMP2.

**Conclusion:**

Ferroptosis-related gene LAMP2 is a potential biomarker with prognostic value for prostate cancer.

## 1. Introduction

Prostate cancer is the second most commonly diagnosed malignancy in men worldwide, with an estimated incidence of 1.4 million in 2020 [1]. Distribution of prostate cancer incidence and lethality follows genetics, environments, lifestyles, and/or interactions between various factors. Prostate cancer is a highly heterogeneous disease with significant difference in prognosis between indolent and aggressive phenotypes, and poor outcomes of aggressive prostate cancer continue to present a major challenge in prostate cancer treatment. Given the critical role of androgens in the progression of prostate cancer, androgen deprivation therapy (ADT) has served as one of the main treatments, while castration resistant prostate cancer usually becomes the ultimate regression of advanced and aggressive prostate cancer with fast progression, multiple metastasis, and poor outcomes [2].

Ferroptosis is an iron-dependent form of regulated cell death driven by excessive lipid peroxidation and has been implicated in the progression and therapeutic responses of various cancerous diseases [3]. On the other hand, ferroptosis-induced damage can trigger inflammation-associated immunosuppression in the tumor microenvironment, thus favoring tumor growth. Ferroptosis is modulated by a series of proteins which served as drivers or suppressors, while gene expression patterns could be altered during the process of carcinogenesis and progression, especially in advanced cancers [4]. Currently, the mechanism by which ferroptosis promotes prostate cancer to acquire aggressive phenotypes, and progress to metastasis or CRPC remains unclear. Based on high-throughput sequencing, it is an effective method to explore crosstalk and mechanisms using a gene expression profile estimated from whole-genome microarray analysis. In our study, we explored differentially expressed genes (DEGs) between localized prostate cancer and CRPC using datasets from Gene Expression Omnibus (GEO) and correlated DEGs with ferroptosis. We aimed to identify potential ferroptosis-related genes in CRPC and further analyzed gene expression associated with clinical characteristics and prognosis to reveal the crosstalk of ferroptosis and CRPC.

## 2. Materials and Methods

### 2.1. RNA Sequencing Data Preparation and Identification of DEGs

We first downloaded RNA sequencing data of localized prostate cancer and metastatic CRPC in the microarray dataset GSE35988 from two platforms. One is Agilent-012391 Whole Human Genome Oligo Microarray, GPL6848, and the other is Agilent-014850 Whole Human Genome Microarray, GPL6480. As results, 59 samples of localized prostate cancer and 35 samples of CRPC were merged for analysis of DEGs [5]. Besides, RNA sequencing data of prostate cancer and paracancerous normal tissue from the Illumina HiSeq RNA sequencing platform correlated with the clinical information of The Cancer Genome Atlas (TCGA) database was also downloaded for analysis.

We first performed different expressed analysis using the online tool GEO2R. We set the adjusted *p* value <0.05 and 2-fold change (|log2FC| ≥ 1) as thresholds for differential expression. We downloaded gene clusters of both drivers and suppressors for ferroptosis from FerrDb V2 and constructed a Venn diagram to extract DEGs correlated with ferroptosis [6].

### 2.2. Enrichment Analysis and Protein-Protein Interaction Networks

We submitted DEGs extracted from the Venn diagram into the online database Metascape for enrichment analysis of Gene Ontology (GO) and Kyoto Encyclopedia of Genes and Genomes (KEGG), including biological process (BP), cellular component (CC), molecular function (MF), and pathways [7]. Accumulative hypergeometric *p* values and enrichment factors were calculated and used for filtering. The remaining significant terms were then hierarchically clustered into a tree based on Kappa-statistical similarities among their gene memberships. Then, 0.3 kappa score was applied as the threshold to cast the tree into term clusters. We selected a subset of representative terms from the full cluster and converted them into a network layout. All protein-protein interactions among input genes were extracted from PPI data sources and formed a PPI network. MCODE algorithm was applied to this network to identify neighborhoods where proteins are densely connected, and then we identified genes densely connected into the networks as hub genes for further analysis.

### 2.3. Correlations between Hub Genes and Clinical Characteristics

We conducted survival analysis with univariate Cox proportional hazard regression by R software for all hub genes associated with overall survival (OS) and disease-specific survival (DSS) using TCGA data of prostate cancer. We set 50% as the cut-off value dividing two groups of low and high expression and merged significant genes in multivariate regression when *p* value <0.1 to evaluate the prognostic value. We generated forest plots to illustrate hazard risk (HR) with 95% confidence interval (CI).

Then, we retrieved prognostic genes in the Human Protein Atlas (HPA) database for RNA expression in various tissues and pan cancers, with immunohistochemical staining to validate gene expression in prostate tissue. Different expression, receiver operating characteristics (ROC), and survival analysis for prognostic genes in different subgroups of prostate cancer would be conducted.

### 2.4. Correlation with Immune Infiltration, MicroRNA Crosstalk, and Methylation

We conducted a single sample gene set enrichment analysis (ssGSEA) for genes with significant prognostic value to analyze correlations with 24 types of immune cells to investigate the immune infiltrates using the GSVA package of R software [8, 9]. The infiltration of immunocytes correlated with gene expression was analyzed by Spearman correlation.

We submitted prognostic genes into the TarBase v.8 database to search for microRNAs in potential regulation, selected 10 top microRNAs ordered by prediction score, and subsequently performed Spearman correlation for microRNAs using TCGA data [10].

We evaluated the methylation levels of prognostic genes in prostate cancer using the UALCAN database and DiseaseMeth database, while we evaluated methylation sites using the MEXPRESS database [11–15].

### 2.5. Statistical Analysis

**Figure 1:**
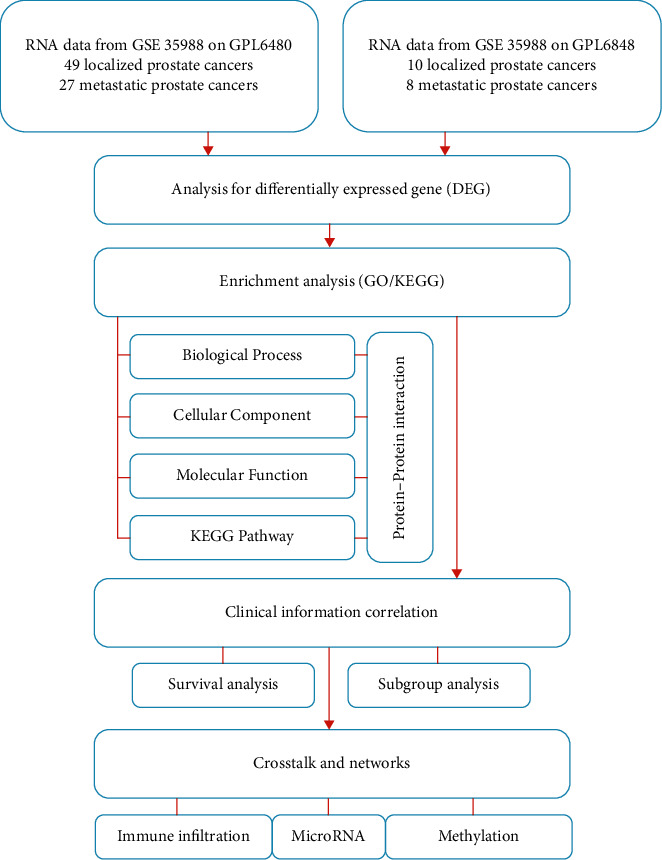
A flow chart of current bioinformatics study.

We performed statistics using GraphPad Prism 8.0 and R software 3.6.3 and presented results in the form of medians with 95% CIs. Two-side *p* value <0.05 was considered as statistical difference if not specifically stated. We summarized the whole process of this study as a flow chart (Figure 1).

## 3. Results

### 3.1. Identification of DEGs in CRPC Correlated with Ferroptosis

**Figure 2:**
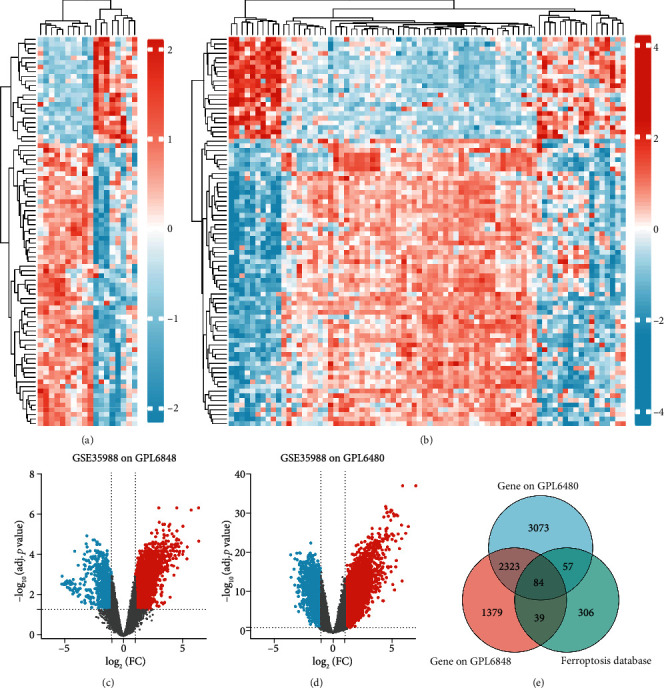
Differential gene expression related to ferroptosis between localized prostate cancer and CRPC. (a) Heat map presenting gene distribution of GSE35988 in GPL6848. (b) Heat map presenting gene distribution of GSE35988 in GPL6480. (c) Volcanic map presenting gene distribution of GSE35988 in GPL6848. (d) Volcanic map presenting gene distribution of GSE35988 in GPL6480. (e) Venn diagram extracting DEGs correlated with ferroptosis between localized prostate cancer and CRPC.

We downloaded RNA sequencing data of GSE35988 and performed differential expression analysis between CRPC and localized prostate cancer. We generated heat maps and volcanic maps presenting gene distribution (Figures 2(a)–2(d)). Subsequently, we downloaded gene clusters of both drivers and suppressors for ferroptosis and constructed a Venn diagram extracting 84 DEGs correlated with ferroptosis (Figure 2(e)).

### 3.2. Enrichment Analysis and Protein-Protein Interaction Networks

**Figure 3:**
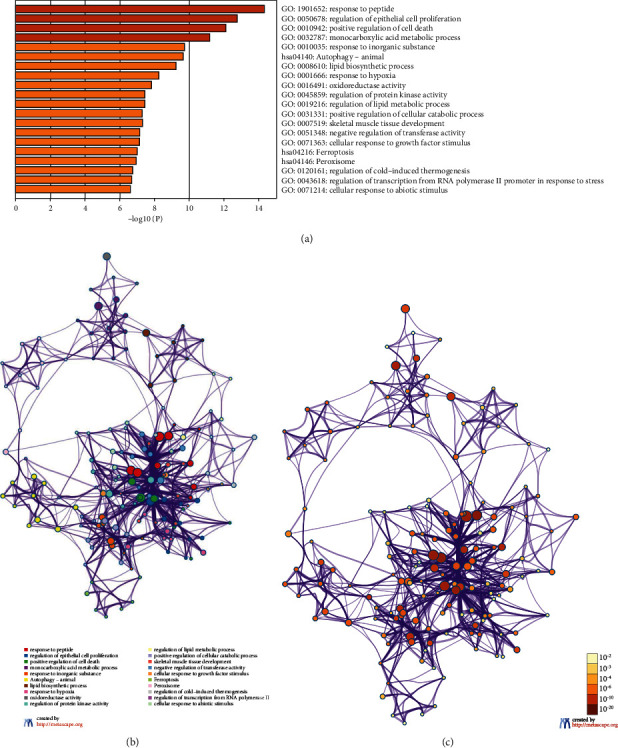
Enrichment analysis for DEGs. (a) Representative terms of enriched ontology clusters. (b) Representative terms in the whole networks and each term represented by a circle node. (c) Nodes representing terms colored by *p* value.

**Figure 4:**
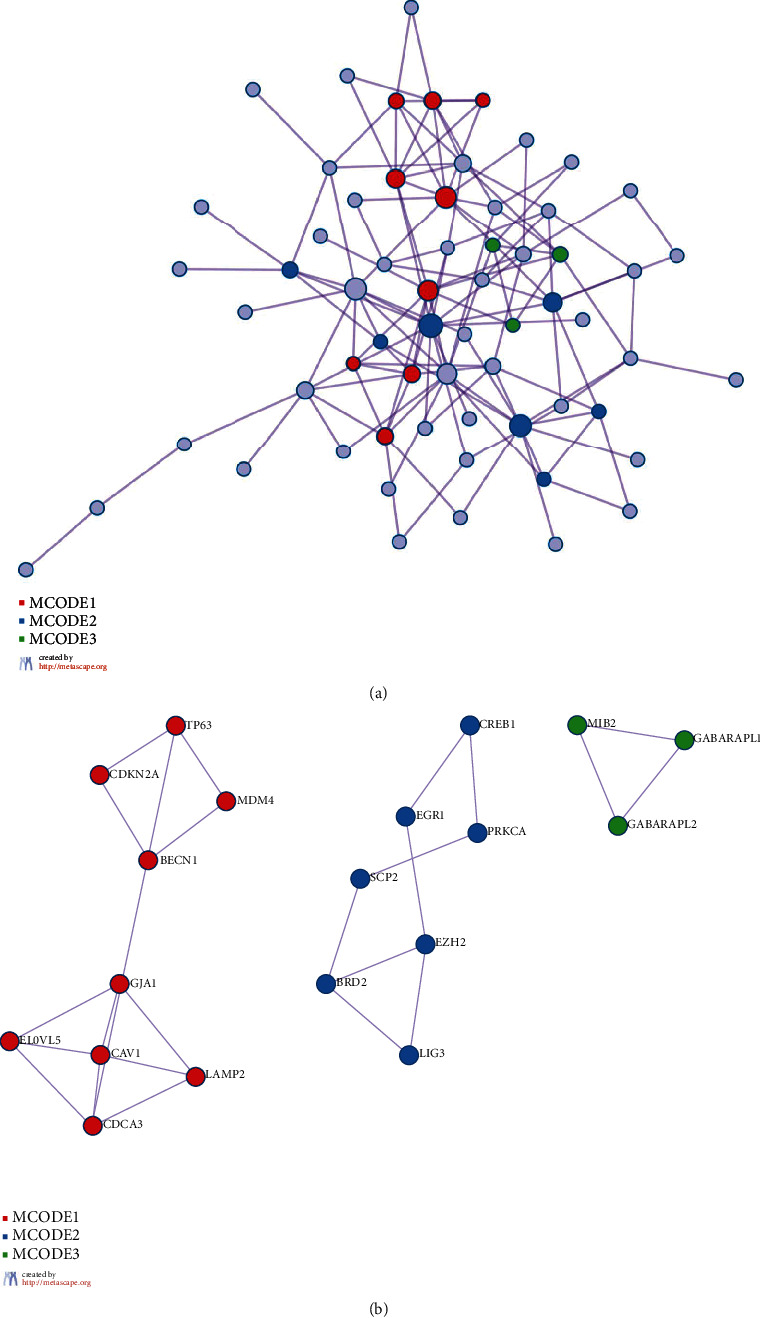
Protein-protein interaction. (a) Protein-protein interactions among DEGs extracted from PPI data source to form a PPI network. (b) MCODE algorithm applied to identify neighborhoods where proteins are densely connected.

We submitted ferroptosis related DEGs to the Metascape database for enrichment analysis including BP, CC, MF, and KEGG pathways. We obtained enriched ontology clusters and presented representative terms (Figure 3(a)). We selected a subset of representative terms from the full cluster and converted them into a network layout. More specifically, each term is represented by a circle node, where its size is proportional to the number of input genes which fall under that term, and its color represented its cluster identity (Figure 3(b)), while the same enrichment network displayed its nodes colored by *p* value (Figure 3(c)). Terms with a similarity score > 0.3 are linked by an edge. Furthermore, all protein-protein interactions among input genes were extracted from PPI data sources and formed a PPI network (Figure 4(a)). MCODE algorithm was then applied to this network to identify neighborhoods where proteins are densely connected (Figure 4(b)). As results, we identified 19 genes densely connected into networks as hub genes for further analysis (Table 1).

### 3.3. Correlation between Hub Genes and Clinical Characteristics

**Figure 5:**
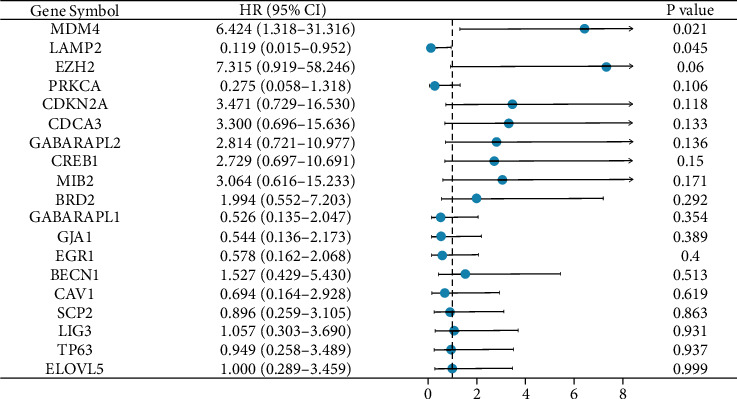
Forest plot presenting Cox regression of overall survival for each hub gene ordered by *p* value.

We conducted survival analysis with univariate Cox proportional hazard regression for all hub genes associated with OS and DSS using TCGA data of prostate cancer. We found that only MDM4 (HR = 6.424, *p* = 0.021) and LAMP2 (HR = 0.119, *p* = 0.045) were significantly associated with OS in prostate cancer, and no significant association was found in the analysis of DSS. We conducted Tables 2 and 3 to describe Cox results and a forest plot showing HRs of each hub gene associated with OS (Figure 5). We further found that LAMP2 was still significantly associated with OS in multivariate Cox regression (HR = 0.095, *p* = 0.028), indicating its prognostic value in prostate cancer.

**Figure 6:**
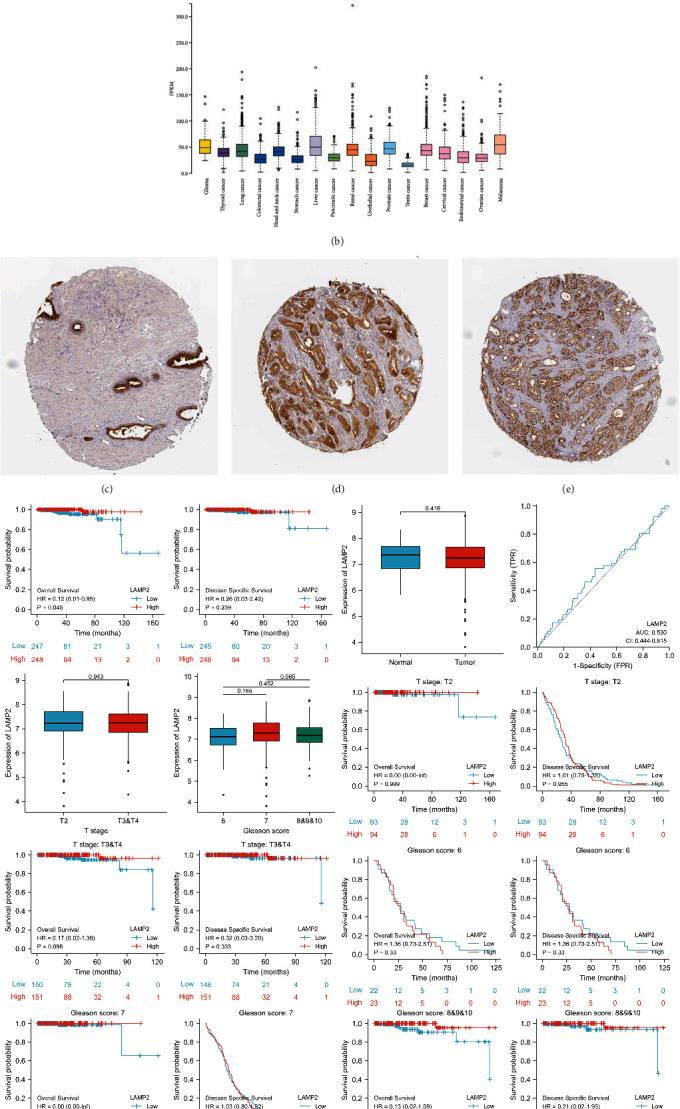
Expression of LAMP2 with clinical information. (a) RNA expression of LAMP2 in normal tissues. (b) RNA expression of LAMP2 in pan cancers. (c) Immunohistochemical staining validated LAMP2 expression in normal prostate tissue. (d) Immunohistochemical staining validated LAMP2 expression in low grade prostate cancer. (e) Immunohistochemical staining validated LAMP2 expression in high grade prostate cancer. (f) Differential expression, ROC analysis, and survival analysis for LAMP2.

Moreover, we retrieved LAMP2 in the HPA database for RNA expression in various tissues and pan cancers (Figures 6(a) and 6(b)). Immunohistochemical staining validated LAMP2 expression in prostate tissue (both normal and cancerous tissues) with cytoplasmic or membranous location (Figures 6(c)–6(e)). Then, we conducted survival analysis for LAMP2 in different subgroups of prostate cancer while no significant association was detected (Figure 6(f)). Besides, we compared differential expression and ROC analysis for other hub genes (Figures S1 and S2).

### 3.4. LAMP2 Correlated with Immune Infiltration, MicroRNA Crosstalk, and Methylation

**Figure 7:**
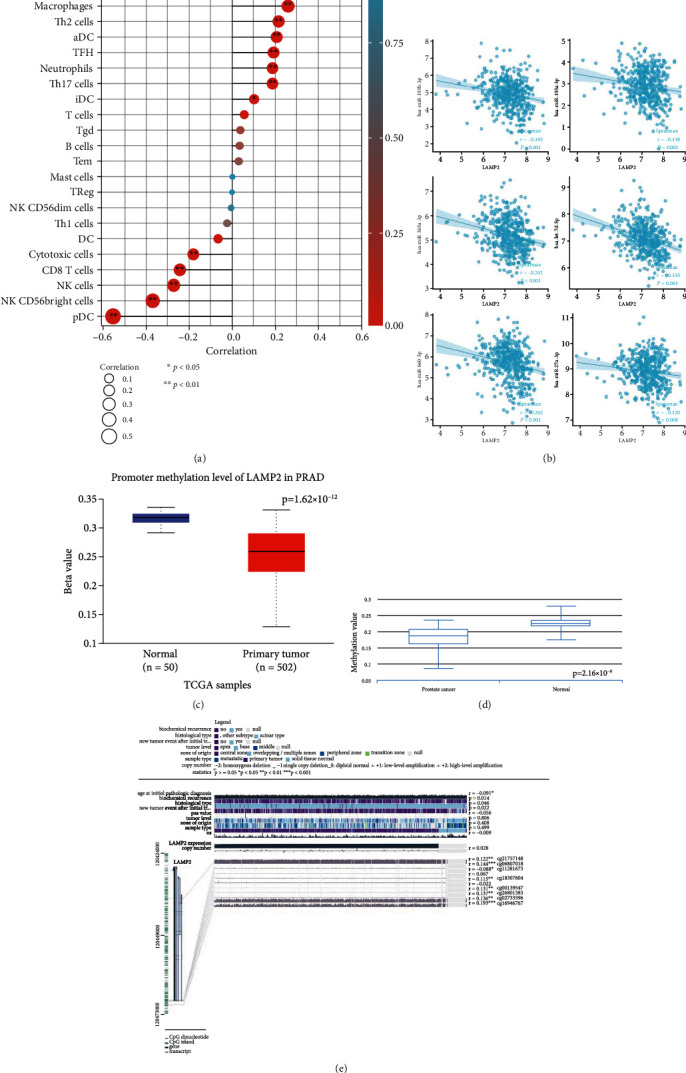
LAMP2 correlated to immune infiltration, microRNA crosstalk, and methylation. (a) LAMP2 expression related to immune cell infiltration. (b) 6 top-correlated microRNAs with LAMP2 expression. (c) Prostate cancer presenting significantly decreased level of LAMP2 in UALCAN database. (d) Prostate cancer presenting significantly decreased level of LAMP2 in DiseaseMeth database. (e) 8 methylation sites associated with LAMP2, noted on the right side.

We employed Spearman correlation to investigate the correlation between LAMP2 expression and immune cell infiltration level using TCGA data quantified as the ssGSEA score and revealed 15 types of immune cells significantly correlated with LAMP2 expression (Figure 7(a)), indicating the vital role of LAMP2 in the immune infiltration in prostate cancer.

We submitted LAMP2 into the TarBase v.8 database to search for microRNAs in potential regulation and selected 10 top microRNAs ordered by the prediction score listed in Table 4. We subsequently performed Spearman correlation for microRNAs with LAMP2 and identified 6 microRNAs with significant correlation (Figure 7(b)).

We evaluated the promoter methylation level of LAMP2 in prostate cancer using the UALCAN database and found prostate cancer presenting a significantly decreased level (Figure 7(c)). Similar results from the DiseaseMeth database revealed a decreased level of methylation in prostate cancer (Figure 7(d)). Additionally, using the MEXPRESS database, we evaluated the methylation sites of DNA sequence and found 8 methylation sites associated with LAMP2 (Figure 7(e)).

## 4. Discussion

Prostate cancer is a highly heterogeneous malignancy, and metastatic CRPC is usually a destination of prostate cancer progressing to terminal stages, accumulating various aggressive cancerous phenotypes and lethal genetic alterations. Ferroptosis is a novel form of programmed cell death, and its regulatory role in tumor development is complex and multifaceted, while there are few studies on ferroptosis in prostate cancer, especially in aggressive cancer or CRPC. Therefore, it is of great interest to investigate ferroptosis associated with progression and prognosis in prostate cancer. In our study, we first analyzed the differential expression of gene profiles between localized prostate cancer and metastatic CRPC from the GEO dataset and obtained an intersection with a cluster of known ferroptosis-related genes, identifying 84 DEGs in CRPC related to ferroptosis. We further identified 19 hub genes densely connected into networks by enrichment analysis. Subsequently, we performed survival analysis and Cox regression for these genes and identified LAMP2 with significantly prognostic values in OS and DSS of prostate cancer. On this basis, we found immune infiltration of various immune cells significantly correlated with LAMP2 expression in prostate cancer. Furthermore, we analyzed and identified multiple microRNAs associated with LAMP2 expression in prostate cancer. In addition, we found that the methylation level of LAMP2 in prostate cancer was significantly associated with cancer and identified eight methylation sites for LAMP2. All of these results suggested that the ferroptosis-related LAMP2 played an important role in progression and prognosis in prostate cancer.

LAMP2, described as lysosomal associated membrane protein 2, located in chromosome X, is one of the most abundant transmembrane proteins of the lysosome, usually highly glycosylated probably forming a continuous glycoprotein layer at the luminal side of the lysosomal membrane, maintain the lysosomal stability and involving in direct transport events across the membrane [16–18]. Deficiency of lysosomal membrane proteins has been shown to cause clinical manifestations ranging from severe visceral symptoms to neurodegeneration, and a classic example associated with null mutation of LAMP2 is Danon disease [19]. Beside, emerging evidence has revealed that lysosomal membrane proteins are inextricably linked to glucose and lipid metabolism, including cholesterol metabolism, lipophagy, and lipoprotein regulation, and this relationship represents a mutual influence and regulation [20]. On the other hand, abnormalities of lipid metabolism are also inextricably linked to the carcinogenesis and progression of prostate cancer. Our previous meta-analysis has revealed the significant association between statin use and biochemical recurrence in prostate cancer patients receiving curative treatment [21]. Taken together, LAMP2-associated abnormalities in lipid metabolism and related signaling networks might play important roles in mechanisms of prostate cancer invasion and progression.

Ferroptosis is an iron-dependent form of programmed cell death caused by unrestricted lipid peroxidation and subsequent plasma membrane rupture [22]. Ferroptosis could be induced through extrinsic or intrinsic pathways, in which iron accumulation and lipid peroxidation are two key signals initiating membrane oxidative damage and ferroptosis [23]. In oncology, cancer cells, which are resistant to conventional therapies or with high propensity of metastasis, might be particularly susceptible to ferroptosis [24, 25]. As a ferroptosis suppressor, LAMP2 was reported with protection against oxidative stress-induced ferroptosis. Inhibiting LAMP2 would decrease cytosolic cysteine concentration, leading to reduced glutathione content, decreased antioxidant capacity, and mitochondrial lipid peroxidation, which in turn triggered oxidative stress-induced ferroptosis [26]. Undertaking the previous context, lipid metabolism-related ferroptosis involved pathways and networks might have important roles in carcinogenesis and progression, which could help to identify biomarkers to stratify malignancy appropriately in clinical treatment and explain how therapies regulating cholesterol metabolism could affect cancer prognosis.

Our current study focused on the difference of gene expression profiles between localized prostate cancer and CRPC and conducted a comprehensive bioinformatics study to investigate the crosstalk and mechanism of progression and metastasis related to ferroptosis in prostate cancer. However, there were still some limitations needed to be taken into consideration. Despite with significant prognostic values in prostate cancer, however, no significant difference of LAMP2 expression between cancerous and normal tissues was detected, and no significant results were found in differential expression and survival analyses in subgroups. In complex signaling networks of ferroptosis, the effect of LAMP2 on prostate cancer could be subject to complex regulation, and these should be corroborated and validated by further experiments. No significant difference was found in the DSS analysis, and considering the extreme heterogeneity of prostate cancer, especially CRPC, various confounding factors might involve and influence the survival outcomes of CRPC. In addition, noncoding RNAs and epigenetic modifications seem to have effects on LAMP2 expression and related ferroptosis signaling networks; however, lacking experimental validation, we required further studies to investigate the regulatory mechanisms.

In conclusion, we compared differentially expressed genes between localized prostate cancer and metastatic CRPC and identified ferroptosis related gene LAMP2 as a potential biomarker with prognostic value for prostate cancer.

## Figures and Tables

**Table 1:** Hub genes related to ferroptosis with differential expression in CRPC.

Gene symbol	Ferroptosis factors	Location	Most expressed tissue
BECN1	Driver	Chromosome17	Skeletal muscle
CDCA3	Driver	Chromosome12	Retina
CDKN2A	Driver	Chromosome9	Pituitary gland
EGR1	Driver	Chromosome5	Ovary
ELOVL5	Driver	Chromosome6	Adipose tissue
GABARAPL1	Driver	Chromosome12	Skeletal muscle
GABARAPL2	Driver	Chromosome16	White matter
GJA1	Driver	Chromosome6	Cerebral cortex
LIG3	Driver	Chromosome17	Testis
MDM4	Driver	Chromosome1	Bone marrow
MIB2	Driver	Chromosome1	Skeletal muscle
PRKCA	Driver	Chromosome17	Hippocampal formation
SCP2	Driver	Chromosome1	Liver
BRD2	Suppressor	Chromosome6	Testis
CAV1	Suppressor	Chromosome7	Adipose tissue
CREB1	Suppressor	Chromosome2	Testis
EZH2	Suppressor	Chromosome7	Bone marrow
LAMP2	Suppressor	ChromosomeX	White matter
TP63	Suppressor	Chromosome3	Skin

**Table 2:** Cox regression for hub genes associated with overall survival of TCGA data.

Gene symbol	Cases	Univariate analysis		Multivariate analysis^∗∗^	
		HR (95% CI)^∗^	*p* value	HR (95% CI)	*p* value
MDM4	495	6.424 (1.318-31.316)	0.021	5.242 (0.780-35.217)	0.088
LAMP2	495	0.119 (0.015-0.952)	0.045	0.095 (0.012-0.771)	0.028
EZH2	495	7.315 (0.919-58.246)	0.06	4.929 (0.572-42.488)	0.147
PRKCA	495	0.275 (0.058-1.318)	0.106		
CDKN2A	495	3.471 (0.729-16.530)	0.118		
CDCA3	495	3.300 (0.696-15.636)	0.133		
GABARAPL2	495	2.814 (0.721-10.977)	0.136		
CREB1	495	2.729 (0.697-10.691)	0.15		
MIB2	495	3.064 (0.616-15.233)	0.171		
BRD2	495	1.994 (0.552-7.203)	0.292		
GABARAPL1	495	0.526 (0.135-2.047)	0.354		
GJA1	495	0.544 (0.136-2.173)	0.389		
EGR1	495	0.578 (0.162-2.068)	0.4		
BECN1	495	1.527 (0.429-5.430)	0.513		
CAV1	495	0.694 (0.164-2.928)	0.619		
SCP2	495	0.896 (0.259-3.105)	0.863		
LIG3	495	1.057 (0.303-3.690)	0.931		
TP63	495	0.949 (0.258-3.489)	0.937		
ELOVL5	495	1.000 (0.289-3.459)	0.999		

^∗^HR = hazard ratio; CI = confidence interval. ^∗∗^Multivariate regression was conducted when *p* value <0.1.

**Table 3:** Cox regression for hub genes associated with disease specific survival of TCGA data.

Gene symbol	Case	Univariate analysis	
		HR (95% CI)^∗^	*p* value
MDM4	495	5.498 (0.611-49.447)	0.128
BRD2	495	4.902 (0.544-44.139)	0.156
CREB1	495	4.569 (0.505-41.305)	0.176
BECN1	495	3.906 (0.436-34.947)	0.223
PRKCA	495	0.262 (0.029-2.412)	0.237
LAMP2	495	0.264 (0.029-2.419)	0.239
GABARAPL1	495	0.285 (0.032-2.567)	0.263
CDCA3	495	3.496 (0.388-31.466)	0.264
GJA1	495	0.286 (0.031-2.627)	0.269
SCP2	495	3.437 (0.383-30.816)	0.27
CDKN2A	495	3.410 (0.373-31.174)	0.277
TP63	495	0.323 (0.035-2.961)	0.318
MIB2	495	3.050 (0.316-29.407)	0.335
CAV1	495	0.357 (0.037-3.442)	0.373
GABARAPL2	495	1.793 (0.298-10.805)	0.524
EGR1	495	0.580 (0.096-3.501)	0.553
ELOVL5	495	1.484 (0.248-8.882)	0.665
LIG3	495	1.437 (0.240-8.605)	0.691
EZH2	495	513316338.157 (0.000-Inf)	0.999

^∗^HR = hazard ratio; CI = confidence interval. ^∗∗^Multivariate regression was refused since no significance was in univariate analysis.

**Table 4:** 10 top microRNAs correlated to LAMP2 expression.

Symbol	Validated tissue	Validated cell line	Predicted score
Hsa-miR-193b-3p	Cervix	HELA	0.897
Hsa-miR-193a-3p	Cervix	HELA	0.888
Hsa-miR-369-3p	Pancreas and brain	BETA	0.823
Hsa-miR-365a-3p	Cervix	HELA	0.812
Hsa-miR-1246	Pleura	BC1	0.751
Hsa-miR-340-5P	Kidney	HEK293	0.65
Hsa-miR-634	Bone marrow	HS5	0.644
Hsa-let-7d-5p	Bone marrow and pancreas	HMSC	0.639
Hsa-miR-660-5p	Pleura	BC1	0.624
Hsa-miR-27a-3p	Umbilical vein	HUVEC	0.615

## Data Availability

We downloaded RNA sequencing data of localized prostate cancer and metastatic CRPC in the microarray dataset GSE35988 from two platforms. One is Agilent-012391 Whole Human Genome Oligo Microarray, GPL6848, and the other is Agilent-014850 Whole Human Genome Microarray, GPL6480. Besides, RNA sequencing data of prostate cancer and paracancerous normal tissue from the Illumina HiSeq RNA sequencing platform correlated with the clinical information of The Cancer Genome Atlas (TCGA) database was also downloaded for analysis.

## Supplementary Materials

Supplementary 1 Figure S1: differential expression of TCGA data for hub genes other from LAMP2.

Supplementary 2 FigureS2. ROC analysis of TCGA data for hub genes other from LAMP2.
