# Supplementary figures and images for: Ferroptosis-Related Prognostic Gene LAMP2 Is a Potential Biomarker Differential Expressed in Castration Resistant Prostate Cancer
Source: Dis Markers. 2023 Jan 20;2023:8295113. doi: 10.1155/2023/8295113 (PMC9893524; doi:10.1155/2023/8295113)

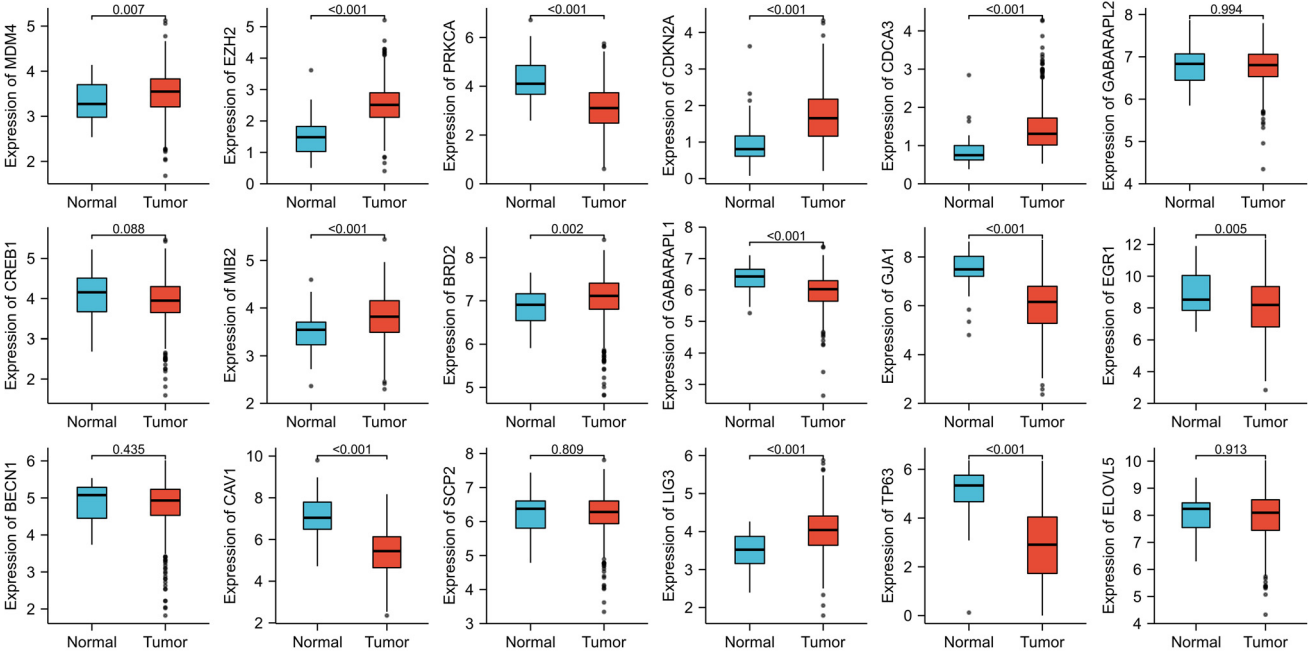

Supplement: Supplementary 1 — Figure S1: differential expression of TCGA data for hub genes other from LAMP2. [file 8295113.f1.pdf]

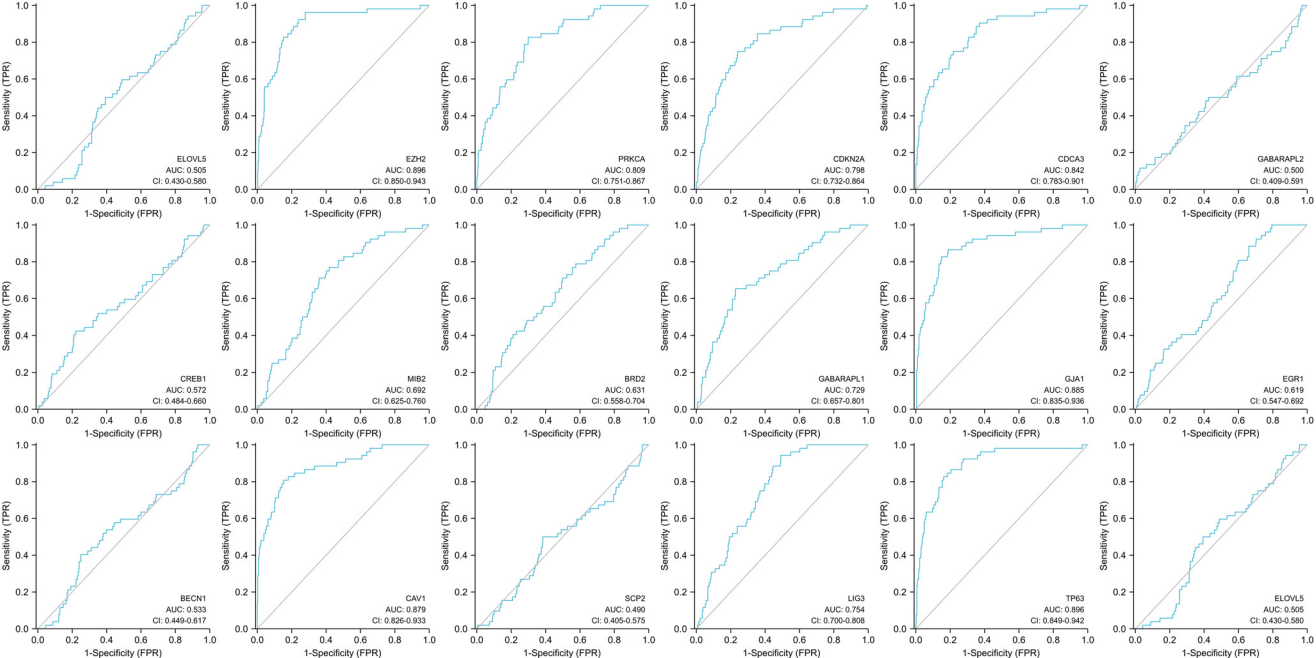

Supplement: Supplementary 2 — FigureS2. ROC analysis of TCGA data for hub genes other from LAMP2. [file 8295113.f2.pdf]
